# Swimming behaviour as an alternative endpoint to assess differences in abiotic stress sensitivities between strains of *Brachionus koreanus* (Rotifera: Monogononta)

**DOI:** 10.1007/s11356-023-26190-3

**Published:** 2023-03-13

**Authors:** Luana Granada, Marco F. L. Lemos, Peter Bossier, Sara C. Novais

**Affiliations:** 1 MARE – Marine and Environmental Sciences Centre & ARNET – Aquatic Research Network, ESTM, Polytechnic of Leiria, 2520-641 Peniche, Portugal; 2 Laboratory of Aquaculture & Artemia Reference Center, Department of Animal Sciences and Aquatic Ecology, Faculty of Bioscience Engineering, Ghent University, 9000 Ghent, Belgium

**Keywords:** Aquaculture, Behavior, *Brachionus koreanus*, Metals, Monogonont rotifer, Swimming capacity

## Abstract

*Brachionus plicatilis* is a cosmopolitan rotifer used as a model organism in several research areas and as live food in aquaculture. Being a species complex, responses to stressors vary even among strains of the same species and, thus, the responses of one species are not representative of the whole complex. This study aimed to address the effects of extreme salinity ranges, and different concentrations of hydrogen peroxide, copper, cadmium, and chloramphenicol, in two strains of *B. koreanus* (MRS10 and IBA3) from *B. plicatilis* species complex, by assessing effects on their survival and swimming capacity. Neonates (0–4 h old) were exposed to the stressors in 48 well-microplates, for 24 and 6 h, to evaluate lethal and behavioural effects, respectively. Tested conditions of chloramphenicol did not show any effects on rotifers. The behavioural endpoint showed to be particularly sensitive to assess the effects of high salinity, hydrogen peroxide, and copper sulfate, as swimming capacity impairment was observed for both strains in the lowest concentrations used in lethal tests. Overall, results showed that IBA3 was more tolerant to the majority of stressors, comparing to MRS10, which may be due to differences in physiological characteristics, highlighting the importance of performing multiclonal experiments. Also, swimming capacity inhibition proved to be a good alternative to the classical lethality tests, being sensitive to lower concentrations and with shorter exposure periods.

## Introduction

As members of marine and freshwater zooplankton communities, rotifers are a relevant group of organisms in aquatic ecosystems connecting phytoplankton and upper trophic levels, while also playing an important role in aquaculture industry as larvae food (Chen et al. 2014; Yúfera 2001). Among rotifers, the monogonont rotifer *Brachionus plicatilis* (Müller, 1786) is one of the best studied taxa and, due to its cosmopolitan distribution in marine habitats, one of the most used in aquaculture (Lubzens 1987).

Rotifers have been also particularly useful for research in different areas given their small size, high ingestion rate, high growth rate, ease of culture in small volumes, short generation time, reproduction mainly via parthenogenesis (genetic homogeneity), and sensitivity to various toxicants (Marcial et al. 2005; Papakostas et al. 2006; Garaventa et al. 2010). Due to these characteristics, in the last decades *B. plicatilis* has been widely used as a model organism in basic research, as well as a bio-indicator and model for ecotoxicology (extensively reviwed by Snell and Janssen 1995; Dahms et al. 2011; Kostopoulou et al. 2012; Rico-Martínez et al. 2013, 2016; Li et al. 2020).

Although several toxicity tests have been developed to assess endpoints such as behaviour, ingestion rates, enzymatic activity, genetic expression, and life table parameters, most of the available studies used 24 h or 48 h lethality tests as preferred endpoints (Rico-Martínez et al. 2016). However, as emphasised by Rebolledo et al. (2020), from a population point of view, chronic tests (e.g. life tables) give a more realistic outcome of the impacts of toxicants in rotifers. On the other hand, these tests require a considerable amount of time, and thus behaviour endpoints have been considered as a viable alternative for their fast assessment of effects (Dahms et al. 2011). A good behavioural response should be specific, sensitive to a wide range of stressors, ecologically relevant, and transversal to different species, which are requirements met by the swimming behaviour of aquatic organisms (Garaventa et al. 2010; Melvin and Wilson 2013). Swimming behaviour has high ecological relevance, since processes such as conspecific recognition, feeding, predator avoidance, and mating when reproducing sexually, are dependent on individual swimming capacity. Therefore, swimming activity assays can give information at a realistic level, since alterations in this endpoint may indirectly predict changes in growth and survival of a population exposed to a contaminant (Chen et al. 2014). Also, due to its sensitiveness to several contaminants at low concentrations, swimming activity has been proposed as a promising tool for toxicity tests in rotifers.

Concerning the susceptibility to toxic substances, large differences have been observed not only among members of the same genus, revealing a need to test several species to assess the effects of contaminants in rotifers (Dahms et al. 2011; Pérez-Legaspi and Rico-Martínez 2001), but also among clones or strains, highlighting the importance of multiclonal experiments (Campillo et al. 2011; Rico-Martínez et al. 2016). This is particularly relevant for *B. plicatilis* since, having once been considered a species with morphological variability, more recent taxonomic and genetic studies revealed it is in fact a species complex (Serra and Fontaneto 2017). Studies have been conducted to better understand the differences among biotypes, addressing parameters such as growth and reproduction (Gómez et al. 1997; Granada et al. 2022; Hagiwara et al. 1995), morphology (Ciros-Pérez et al. 2001; Hwang et al. 2013), lifespan (Gribble et al. 2014; Kaneko et al. 2011; Snare et al. 2013), response to toxicants (Han et al. 2023; Kang et al. 2019), and others.

Thus, the main objective of this work was to evaluate the sensitiveness of the swimming capacity endpoint to assess differences in the response between two strains of *B. koreanus* (from *B. plicatilis* species complex) to a range of different abiotic stressors, including extreme abiotic conditions (low and high salinity), two metals (copper and cadmium), and substances used in aquaculture for disinfection processes (hydrogen peroxide) and to obtain axenic cultures (chloramphenicol).

## Material and methods

### Rotifer cultures

Clone cultures of two strains, MRS10 and IBA, of monogonont rotifer *B. koreanus* were used in this study. Species identification was confirmed by analysis of 16S rRNA genome sequences (Granada et al. 2022).

Cultures were constituted only by parthenogenetic females, reducing the intraspecific variability in their response to stressors. Cultures were maintained at 25 psu (autoclaved artificial seawater, ASW, Instant Ocean Sea Salt with deionized water), room temperature of 25 ± 1 °C, under constant light intensity of 34 µmol m^−2^ s^−1^ (cool white tube lights). Cultures were fed daily with *Tetraselmis* sp. at a final concentration of 10^5^ cells mL^−1^ (Granada et al. 2022).

### Chemicals

Hydrogen peroxide (30%; H_2_O_2_; Merck KGaA, Darmstadt, Germany), copper sulfate (CuSO_4_.5H_2_O; 249.68 g mol^−1^; Alfa Aesar, Ward Hill, MA, USA; 99% purity), cadmium chloride (CdCl_2_; 183.32 g mol^−1^; Merck, Darmstadt, Germany; purity ≥ 99.99%), and chloramphenicol (C_11_H_12_C_l2_N_2_O_5_; 323.13 g mol^−1^; Merck, Darmstadt, Germany; purity ≥ 98%) were used according to the instructions of the manufacturer. Highly concentrated stock solutions were prepared in ASW or Milli-Q water and diluted in ASW to prepare the test solutions with different concentrations. The same stock solution was used to test each strains’ responses to a given stressor. Ethanol was used as solvent to prepare the stock solution of chloramphenicol.

### Exposure setup

The effects of salinity, hydrogen peroxide, copper sulfate, cadmium chloride, and chloramphenicol were assessed on the two strains of rotifers (MRS10 and IBA3), addressing survival and behaviour endpoints. Every assay was performed in 48-well microplates, using 0–4 h old neonates of clone cultures, and consisting of 500 µL of medium per well, where six treatments of a given stressor were tested with six replicates of five rotifers per treatment. Tests were conducted in a climatic room at 25 ± 1 °C, with no light, and rotifers were not fed during exposures.

#### Lethality tests

The acute toxic effects were evaluated following the procedures described in ISO 19820: 2016(E). Briefly, newly hatched neonates (0–4 h old) were exposed to every single stressor for 24 h. Neonates of both strains were challenged with low (0, 0.5, 1.0, 1.5, 2.0, 2.5 psu) and high (60, 65, 70, 75, 80, 85 psu) salinity conditions, hydrogen peroxide (0, 2.21, 2.33, 2.45, 2.58, 2.72 mg L^−1^), copper sulfate (0, 0.50, 0.78, 1.22, 1.92, 3.00 mg L^−1^), cadmium chloride (0, 30.00, 60.62, 122.47, 247.46, 500.00 mg L^−1^), and chloramphenicol (0, 1.00, 3.16, 10.00, 31.62, 100 mg L^−1^). For chloramphenicol, a solvent control was used consisting of ASW with 0.20% ethanol (v/v), corresponding to the maximum solvent concentration used in the chloramphenicol treatments (highest treatment — 100 mg L^−1^).

At the end of the experiment, the number of live rotifers was determined, and the concentration causing 50% lethality (LC_50_) was calculated for each stressor and respective strain. Rotifers were considered dead if not showing any movement during 10 s of observation. Tests were considered valid if the percentage of mortality in the negative control was not higher than 10% (ISO 19820: 2016).

#### Behaviour response

Behaviour effects were evaluated by analysing the swimming capacity of organisms after the exposure to the above-mentioned stressors.

**Fig. 1:**
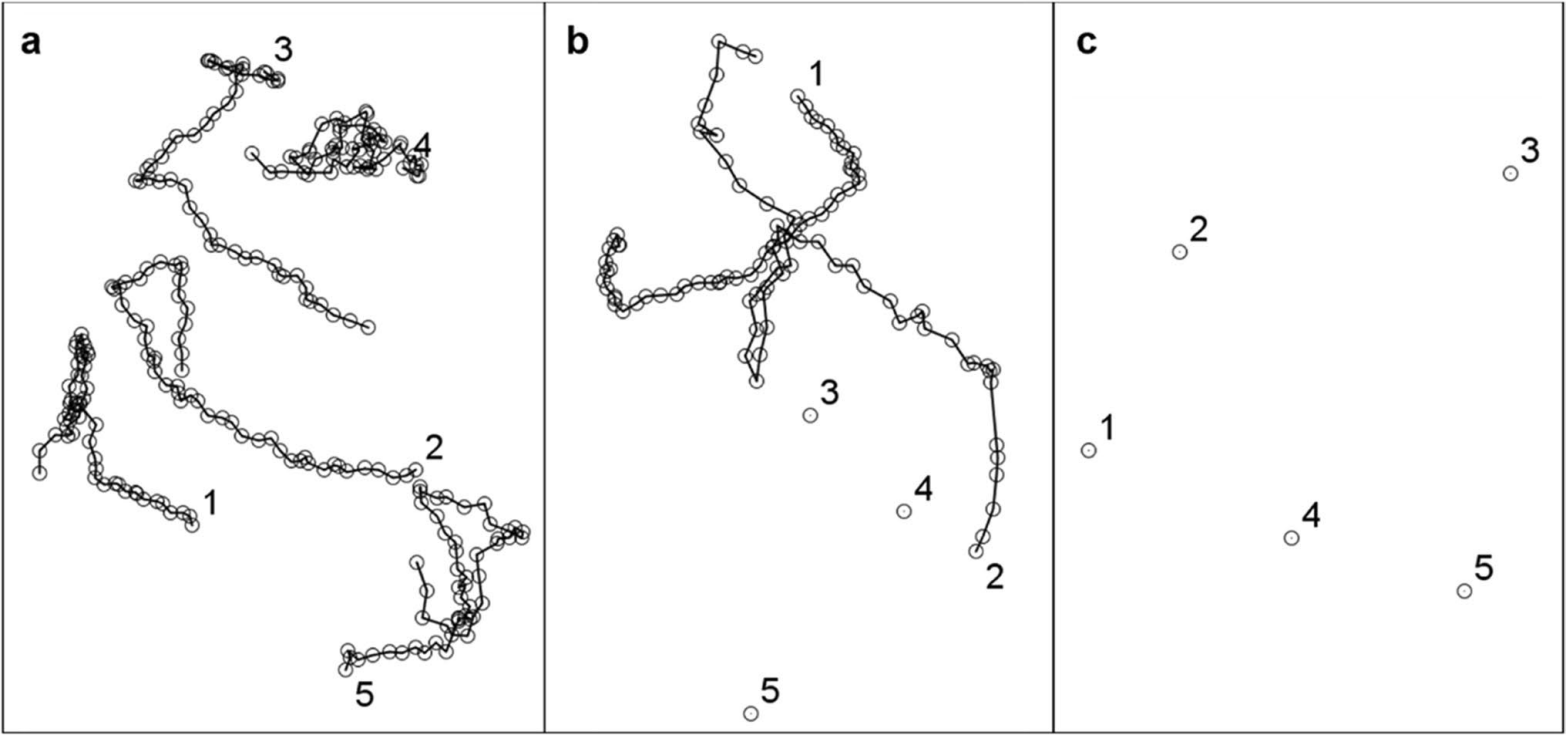
Example of trajectories obtained and analysed when rotifers were exposed to (**a**) control condition, (**b**) middle condition, and (**c**) maximum condition of a stressor. These trajectories were obtained from the exposure of MRS10 neonates to hydrogen peroxide (0 mg L^−1^, 0.87 mg L^−1^, and 2.21 mg L^−1^, respectively)

Newly hatched neonates were exposed to the same concentrations of the lethality tests for 6 h to assess the effect of stressors on the swimming speed, according to Liang et al. (2018). As movement impairment was observed in the lethality tests already in the lower concentration tested for high salinity conditions, hydrogen peroxide, and copper sulfate, a lower range was used to perform the behaviour tests (high salinity: 40, 45.95, 52.78, 60.63, 69.64, 80 psu; hydrogen peroxide: 0, 0.34, 0.54, 0.87, 1.38, 2.21 mg L^−1^; copper sulfate: 0, 0.10, 0.19, 0.36, 0.68, 1.30 mg L^−1^). At the end of the experiment, and before the observation, the volume of each well was reduced to 150 µL to avoid up and down movement in the water column. Each well was recorded for 1 min under a magnifying glass, using the camera AxioCam (Zeiss Microscopy) with 1.4 MP of resolution, connected to the ZEN software (Zeiss Microscopy). All movies were exported under AVI uncompressed format (.avi) and, for each rotifer, trajectories were manually analysed and mean speed calculated, using the image processing plugin MTrackJ, of ImageJ software (Schneider et al. 2012; Yúfera et al. 2005) (Fig. 1). These values were used to calculate the swimming inhibition rate normalized to the average values in control treatment, by the equation: Inhibition rate (%) = [(V_control_ – V_treatment_)/V_control_] × 100 (Chen et al. 2014), where V_control_ is the average values of swimming velocity in the control treatment of a stressor, and V_treatment_ is the swimming velocity in the treatments where rotifers were exposed to that stressor. Values of inhibition rate were used to calculate the swimming capacity of rotifers, by the equation: Swimming capacity (%) = 100 – Inhibition rate, considering a swimming capacity under control conditions of 100%.

Tests were considered valid if the percentage of organisms with swimming capacity impairment in the negative control was not higher than 20%.

### Statistical analysis

In order to evaluate acute and behaviour effects of each stressor on both strains, 24 h-LC_50_ (50% reduction in survival compared to control treatments) and 6 h-EC_50_ (50% reduction in swimming speed compared to control treatments) values were analysed. Firstly, LC_50_ and EC_50_ values, and respective 95% confidence intervals, were calculated based on log concentrations by fitting four-parameter logistic dose–response curves (Y = Bottom + (Top–Bottom)/(1 + 10^(LogEC50−X)*HillSlope)^), where “Y” is the response (% survival for LC_50_ estimation; % swimming capacity for EC_50_ estimation); “Bottom” the basal response (0%), “Top” the maximal response (100%), “X” the logarithm of concentration, and “HillSlope” the slope of the logistic curve. Secondly, to compare the concentrations of effect for each stressor between strains, a global fitting (extra sum of squares *F*-test) was performed. All these analyses were done using GraphPad Prism version 6.00 for Windows (GraphPad Software, La Jolla, CA, USA).

Exceptionally, as in behaviour tests both strains showed a hormetic response to hydrogen peroxide, and MRS10 to cadmium chloride, the EC_50_ values in these cases were calculated based on the model: EC50 = (t*(1 + h*Conc))/(1 + ((0,5 + h*Conc)/0.5)*(Conc/x)^b^), where “t” is the control response, “h” the hormetic effects (estimated between 0.1 and 1), “Conc” the exposure concentration, and “b” a scale parameter (estimated between 1 and 4). These data were analysed using STATISTICA software (StatSoft, Inc., Tulsa, OK, USA).

To assess differences between average velocities in control treatments between strains, independent *T*-tests were performed using the IBM SPSS Statistics 26. Results are presented as mean ± SD. For all statistical tests, the significance level was set at *P* ≤ 0.05.

All graphics were made using GraphPad Prism version 6.00 for Windows.

## Results

**Fig. 2:**
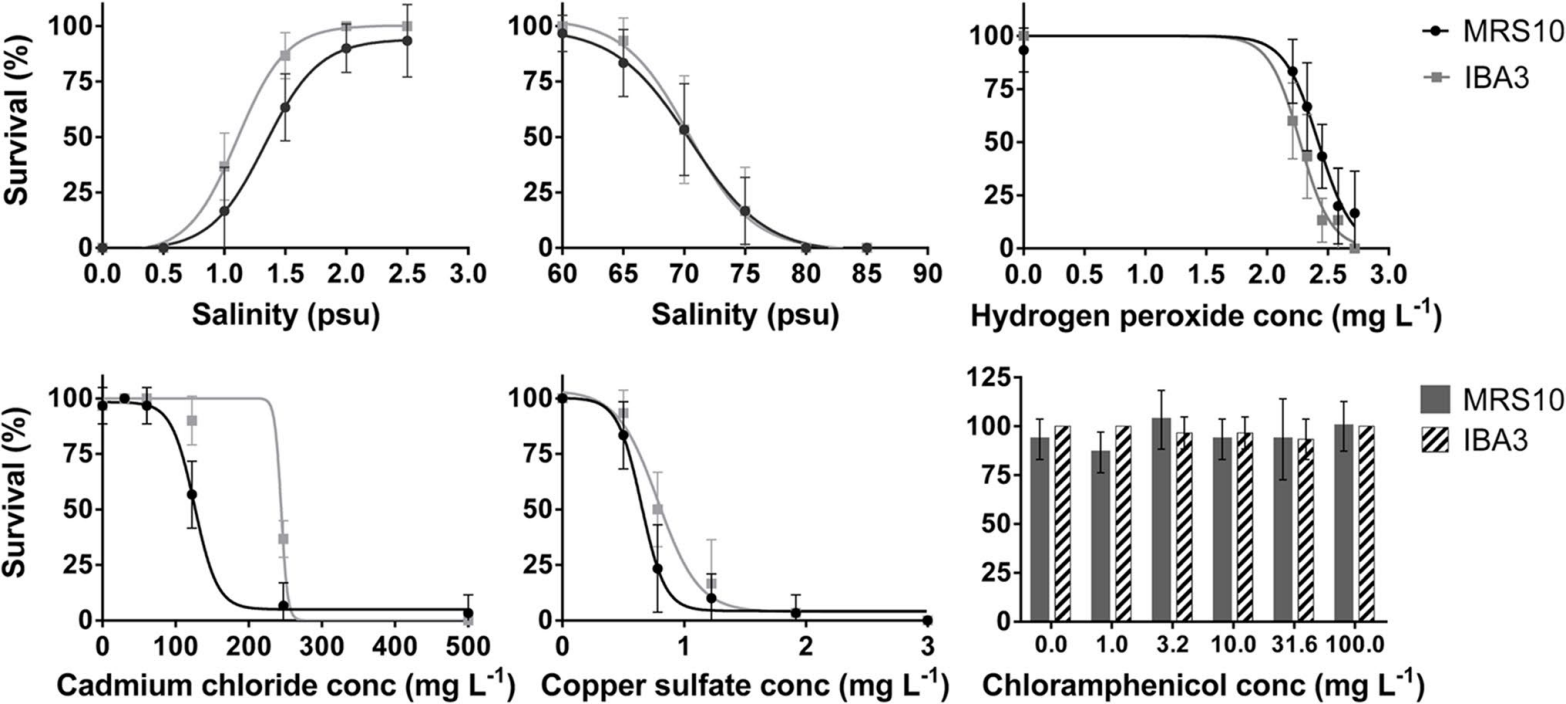
Dose–response curves for effects of exposure to several abiotic stressors on survival of MRS10 and IBA3 strains from *Brachionus plicatilis* species complex

All lethality tests fulfilled the validity criteria as described by the standard guideline (ISO 19820: 2016). Survival results after exposure to low and high salinity, hydrogen peroxide, copper sulfate, cadmium chloride, and chloramphenicol can be seen in Fig. 2, for both strains. The 24 h-LC_50_ values, along with the global fitting comparisons between strains and respective statistical results are given in Table 1.

**Table 1:** Acute LC_50_ values determined for MRS10 and IBA3 neonates, from *Brachionus plicatilis* species complex, after being exposed to several stressors, calculated by fitting four-parameter logistic dose–response model. Strains’ LC_50_ values were compared by global fitting (*P* ≤ 0.05). Values in bold represent the LC_50_ obtained for the more tolerant strain, when statistically significant differences were observed between strains

	MRS10				IBA3				Global fitting	
	Hill slope	24 h LC_50_	LC_50_—95% CI	Adj. *R*^2^	Hill slope	24 h LC_50_	LC_50_—95% CI	Adj. *R*^2^	F (DFn, DFd)	*P*-value
Low salinity (psu)	5.27	1.35	1.26–1.45	0.92	6.21	**1.09**	1.05–1.16	0.98	16.18 (2,68)	< 0.0001
High salinity (psu)	− 23.82	70.13	69.20–71.07	0.92	− 29.11	70.47	69.59–71.37	0.92	0.5761 (2,68)	0.813
Hydrogen peroxide (mg L^−1^)	− 17.47	**2.42**	2.37–2.46	0.77	− 18.54	2.27	2.22–2.32	0.85	12.87 (2,68)	< 0.0001
Copper sulfate (mg L^−1^)	− 5.801	0.6496	0.6068–0.6954	0.93	− 4.412	**0.8055**	0.7485–0.8668	0.93	10.26 (2,68)	0.0001
Cadmium chloride (mg L^−1^)	− 4.148	130.8	121.7–140.6	0.96	− 3.719	**210.5**	195.9–226.1	0.96	36.69 (2,68)	< 0.0001
Chloramphenicol (mg L^−1^)	-	> 100	-	-	-	> 100	-	-	-	-

Concerning the exposure to low salinity conditions, IBA3 showed to be more tolerant with significantly lower LC_50_ (1.09 psu; F_2,68_ = 16.18, *P* < 0.0001) than MRS10 (1.35 psu). At high salinity conditions, MRS10 and IBA3 did not show statistically significant differences, with LC_50_ values of 70.13 psu and 70.47 psu, respectively. MRS10 were more tolerant to hydrogen peroxide, with a significantly higher LC_50_ (2.42 mg L^−1^; F_2,68_ = 12.87, *P* < 0.0001) in comparison with IBA3 (2.27 mg L^−1^). MRS10 showed to be more sensitive to copper sulfate, with a significantly lower LC_50_ (0.65 mg L^−1^; F_2,68_ = 10.26, *P* = 0.0001) when compared with IBA3 (0.81 mg L^−1^). MRS10 was more sensitive to cadmium chloride as well, with a significantly lower LC_50_ (130.8 mg L^−1^; F_2,68_ = 36.69, *P* < 0.0001) when compared with IBA3 (210.5 mg L^−1^). For chloramphenicol it was not possible to calculate LC_50_ values, as the concentrations used (0–100 mg L^−1^) did not have any effects on both strains survival. For this stressor, no statistically significant differences were found between control and solvent control for both lethality and behavioural tests (*P* < 0.05).

**Fig. 3:**
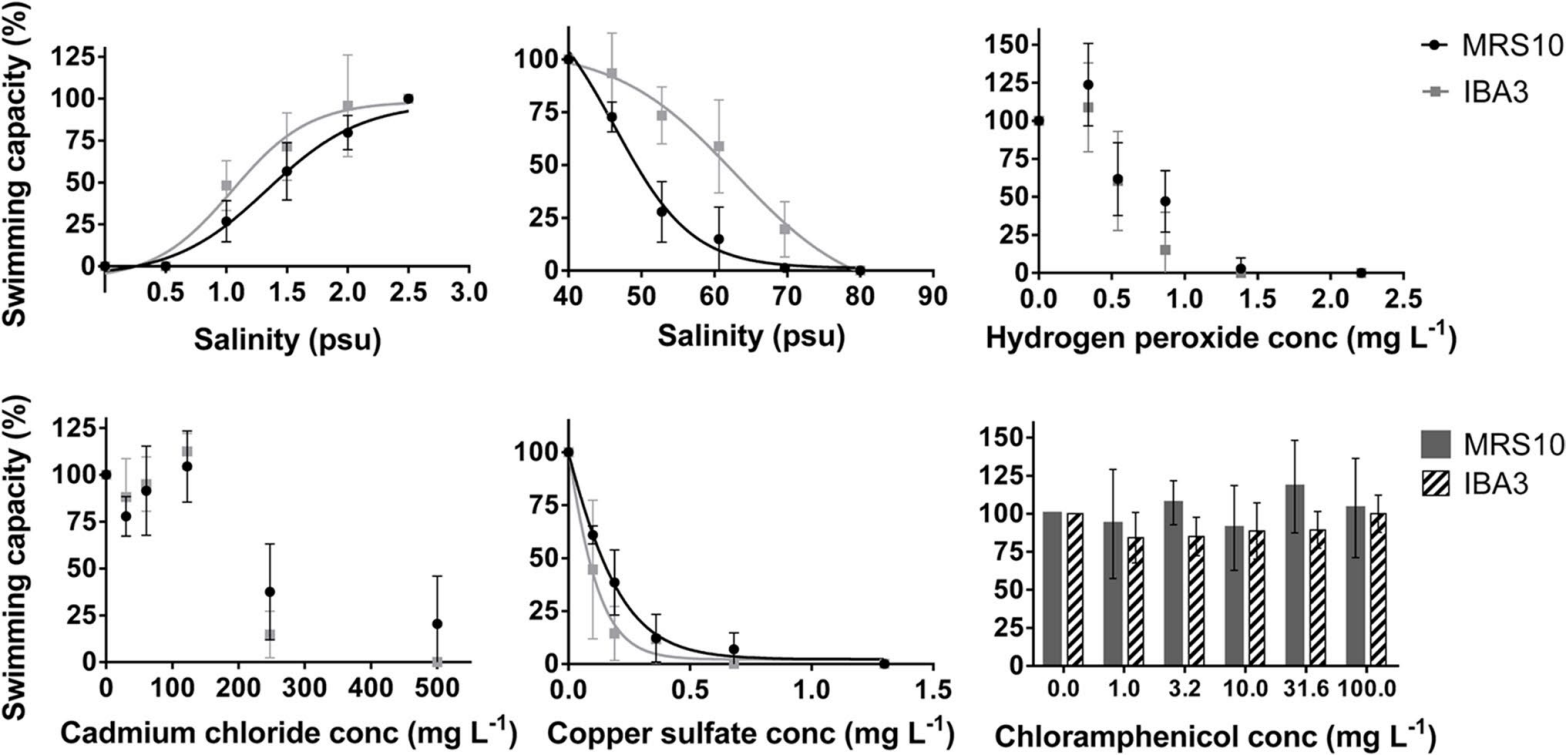
Dose–response curves for effects of exposure to several abiotic stressors on swimming capacity of MRS10 and IBA3 strains from *Brachionus plicatilis* species complex

All behaviour tests fulfilled the established validity criteria. Swimming capacity results after exposure to low and high salinity, hydrogen peroxide, copper sulfate, cadmium chloride, and chloramphenicol can be seen in Fig. 3, for both strains. The 6 h-EC_50_ values, along with the global fitting comparisons between strains and respective statistical results are given in Table 2.

**Table 2:** EC_50_ values concerning inhibition in swimming capacity, determined for MRS10 and IBA3 neonates, from *Brachionus plicatilis* species complex, after being exposed to several stressors, calculated by fitting four-parameter logistic dose–response model or (*) hormetic model. Strains’ EC_50_ values were compared by global fitting (*P* ≤ 0.05). Values in bold represent the EC_50_ obtained for the more tolerant strain, when statistically significant differences were observed between strains

	MRS10				IBA3				Global fitting	
	Hill slope	6 h EC_50_	EC_50_—95% CI	Adj. *R*^2^	Hill slope	6 h EC_50_	EC_50_—95% CI	Adj. *R*^2^	F (DFn, DFd)	*P*-value
Low salinity (psu)	3.584	1.363	1.274–1.459	0.92	3.750	**1.068**	0.942–1.212	0.85	6.753 (2,58)	0.0023
High salinity (psu)	− 11.94	49.55	48.44–50.68	0.90	− 9.757	**60.98**	58.72–63.32	0.84	42.96 (2,58)	< 0.0001
Hydrogen peroxide (mg L^−1^)	-	0.66*	0.48–0.85	0.92	-	0.59*	0.46–0.71	0.91	2.633 (2,58)	0.0805
Copper sulfate (mg L^−1^)	− 1.735	**0.1341**	0.1177–0.1527	0.90	− 1.959	0.0877	0.0639–0.1205	0.67	6.848 (2,58)	0.0021
Cadmium chloride (mg L^−1^)	-	272.14*	190.93–353.34	0.82	− 23.86	230.10	-	0.91	4.639 (2,46)	0.0146
Chloramphenicol (mg L^−1^)	-	> 100	-	-	-	> 100	-	-	-	-

The average swimming velocities in control treatments for MRS10 and IBA3 were 138.00 ± 68.34 and 150.67 ± 57.47 µm sec^−1^, respectively, not showing statistically significant differences (*P* = 0.735). These velocities represent the normal swimming capacity of rotifers used in this study.

For low salinity, as verified for the acute lethality effects, IBA3 was more tolerant than MRS10 (F_2,58_ = 6.753, *P* = 0.0023), with an EC_50_ value of 1.068 psu in comparison with 1.363 psu for MRS10. Regarding high salinity, IBA3 was also more tolerant than MRS10 (F_2,58_ = 42.96, *P* < 0.0001), with an EC_50_ value of 60.98 psu, comparing with 49.55 psu for MRS10. For hydrogen peroxide, no statistically significant differences between strains were observed (F_2,58_ = 2.633, *P* = 0.0805), with MRS10 showing an EC_50_ value of 0.66 mg L^−1^ and IBA3 of 0.59 mg L^−1^. Contrary to the acute test, MRS10 showed to be more tolerant to copper sulfate, with a significantly higher EC_50_ (0.1341 mg L^−1^; F_2,58_ = 6.848, *P* = 0.0021) when compared with IBA3 (0.0877 mg L^−1^). The range of concentrations used in the cadmium exposures showed to affect the swimming capacity of rotifers, and the EC_50_ values were calculated as 272.14 mg L^−1^ and 230.10 mg L^−1^, for MRS10 and IBA3, respectively, being MRS10 more tolerant (F_2,46_ = 4.639, *P* = 0.0146). While both strains showed a logistic response to the overall stressors, a hormetic response was observed for hydrogen peroxide for both strains, and for cadmium chloride for MRS10 strain, under the tested concentrations. As for chloramphenicol, and similarly to the observed for the acute effects, no alterations on the swimming capacity of both strains were verified, considering the tested concentrations.

## Discussion

In this study, the susceptibility of two strains of the *B. plicatilis* species complex, identified as *B. koreanus*, to extreme ranges of salinity, two metals (one essential and one non-essential), one peroxide compound, and one antibiotic, was compared by addressing lethal and behavioural endpoints.

### Differences in sensitivity between strains — effects on survival

Overall, IBA3 showed higher tolerance than MRS10 to the tested stressors in terms of survival, except for hydrogen peroxide, to which it was more sensitive than MRS10, and for high salinity, to which there were no differences between strains.

In the aquatic environment, where organisms are immersed in a liquid and dependent of the medium characteristics, salinity is one of the most important abiotic factors, affecting biological processes from the organism to the ecosystem level (Lee et al. 2017). Seasonal and spatial fluctuations in salinity significantly affect rotifer abundance by changing its growth and fecundity, and regulates competition between species in natural habitats (Kim et al. 2020). In general, rotifer *B. plicatilis* is euryhaline, naturally occurring at salinities between 2 and 65 psu, and being able to tolerate a wider salinity range (1 to 97 psu) (Fielder et al. 2000; Han and Lee 2020; Lowe et al. 2005). However, it is important to notice that, being a species complex, it comprises species and strains with different optimal salinities that, along with other abiotic and biotic factors, mediate their coexistence (Montero-Pau et al. 2011). In the present study, newly hatched neonates were exposed to low and high salinities, with no observed mortality at 2.5 and 60 psu for both strains. Although the results for low salinity were expected, MRS10 and IBA3 appeared to be more tolerant to high salinities than other studies with *B. plicatilis* where total mortality of neonates was verified at 39 and 40 psu (Han and Lee 2020; Joshi 1988). The LC_50_ values (Table 1) obtained at high salinity for both strains (70.13 and 70.47 psu) were however more consistent with the 68.1 psu observed by Yoon and Park (2011) for *B. plicatilis* neonates exposed to brine discharges by desalination industries.

As aquatic organisms, rotifers are also often exposed to chemical products resultant from anthropogenic activities, industry, agriculture, and aquaculture (Jeong et al. 2019). Toxicity studies with several rotifer species, mainly from freshwater environments, have been conducted with a wide range of inorganic (metals and metalloids) and organic compounds (e.g. pesticides, detergents, solvents, colorants), and pharmaceuticals (Rico-Martínez et al. 2016). In this study, the effects of the metals copper sulfate (recommended as reference substance in ISO 19820: 2016) and cadmium chloride were assessed for comparison purposes between strains. Copper is considered an essential metal due to its biological function (Jeong et al. 2019) since, at low concentrations, is necessary for several enzymatic and molecular functions; however, at higher concentrations can be cytotoxic and, in rotifers, has been shown to cause high mortality and abnormal behaviour (Cooper et al. 2014; Han et al. 2013). The 24 h-LC_50_ values observed for MRS10 and IBA3 (0.65 mg L^−1^ and 0.81 mg L^−1^, respectively; Table 1) were within the interval observed in other studies with *B. koreanus* (0.13–1.2 mg L^−1^) (Han et al. 2013; Jung and Lee 2012; Lee et al. 2016), although MRS10 was more sensitive than IBA3. Contrarily to copper, cadmium is a non-essential metal and presents high toxicity at low concentrations (Kang et al. 2018), being very dispersed in the aquatic environment and, due to its high bioavailability, it is easily transferred along the food chain. IBA3 was significantly more tolerant to cadmium than MRS10 (210.5 mg L^−1^ and 130.8 mg L^−1^, respectively) (Table 1). In a study by Kang et al. (2019), the LC_50_ value obtained for neonates of *B. koreanus* (142.69 mg L^−1^) was similar to the one obtained for MRS10 but for only 6 h of exposure. This seems to indicate that MRS10 and IBA3 have higher tolerance to cadmium than the organisms studied by Kang et al. (2019). These authors observed that body size correlated inversely with metabolic rate, which is a key factor for cadmium bioaccumulation. They also showed a body size-dependent interspecific tolerance, with the smaller *B. rotundiformis* presenting the lowest 6 h-LC_50_ value and the larger *B. plicatilis* the highest (123.33 and 309.34 mg L^−1^, respectively) when exposing neonates. Also, for *B. koreanus*, the LC_50_ value increased from 142.69 mg L^−1^ in neonates to 306.07 mg L^−1^ when exposing adults. In fact, IBA3 present larger body length than MRS10 during the initial developmental stages (Granada et al. 2022), which can explain its higher tolerance to this stressor.

Although prohibited in many countries for its adverse health effect in humans, the antibiotic chloramphenicol is still used in aquaculture and animal farming in some developing countries, due to its broad-spectrum antimicrobial activity and low cost (Romero-Soto et al. 2018). It is also used in antibiotic cocktails to eliminate rotifer-associated bacteria and obtain axenic cultures (Martínez-Díaz et al. 2003). Besides antibiotics, hydrogen peroxide is a therapeutant used to prevent and control mortality caused by external fungal infections in cultured fish and their eggs, having the potential to also control external bacterial infections and parasitic infections. The use of hydrogen peroxide in aquaculture and its effects on freshwater and brackish water organisms was reviewed by Schmidt et al. (2006). In mass productions, rotifers grow in association with a complex bacterial ecosystem, and these bacteria can improve the culture by constituting food for rotifers or producing compounds (e.g. vitamin B12) for growth support. However, these bacteria can be harmful for fish larvae, compromising their performance and survival (Martínez-Díaz et al. 2003). Therefore, it is recommended to eliminate the rotifer-associated bacteria before using rotifers as live food. In addition, axenic rotifers are particularly useful for nutritional science, probiotic, molecular biology, development of immune system, and genetic studies (Suga et al. 2011). In this study, when exposed to hydrogen peroxide, MRS10 were more tolerant than IBA3 (LC_50_ of 2.42 mg L^−1^ and 2.27 mg L^−1^, respectively), contrarily to what was observed for the other stressors (Table 1). In another study, exposure to 3% hydrogen peroxide for 6–10 min showed to be effective in eliminating rotifer-associated bacteria in *B. plicatilis*, although rotifer mortality and unviability of amictic eggs were also observed at these conditions (Martínez-Díaz et al. 2003). Concentrations much lower than 0.1% were extremely harmful for both strains in the present study.

Regarding chloramphenicol, no effects on survival were observed for any of the two strains of *B. koreanus*, even at the higher concentration tested (100 mg L^−1^). Martínez-Díaz et al. (2003) have also observed no effects on survival of *B. plicatilis* when the rotifers were exposed to an antibiotic cocktail, with chloramphenicol at a maximum concentration of 40 mg L^−1^. Although no effects on survival were seen in those studies, Suga et al. (2011) showed that when exposing *B. plicatilis* to 40 mg L^−1^ of chloramphenicol, the antibiotic had a toxic effect on germ cell and egg development, along with innate malformations in F2 generation and abnormal sterile neonates. This highlights the increasing importance of studying sub-lethal effects of a substance since the non-existence of lethal effects does not necessarily imply it will not be harmful at other levels.

### Swimming behaviour as a more sensitive endpoint for effect assessment between strains

The movement of *Brachionus* spp. comprises periods of free swimming and periods of attachment to substrata with the pedal gland (Kim et al. 2020). The helical swimming, characteristic of rotifers, results from the coordinated beat of cilia that is controlled by two innerved muscles located in the infraciliature (Charoy and Janssen 1999). For rotifers, swimming is a high energy demand action, highly affected by intrinsic (e.g. female age and body size) and extrinsic factors (Yúfera et al. 2005). Since metabolic activity can compromise cilia beating ability and/or its coordination, leading to alterations in the swimming pattern, studies have been using swimming speed as a parameter to evaluate the physiological condition of rotifers and the status of rotifer mass cultures (Kim et al. 2020; Korstad et al. 1995). A decrease in not only swimming speed but also in swimming frequency is usually observed in rotifers under physical stress, namely when the organisms are trying to maintain the osmotic equilibrium, given that the attachment may be a way to conserve energy and help rotifers to cope with stressful conditions (Kim et al. 2020). In the present work, the endpoint studied was the swimming capacity inhibition by measuring rotifer swimming speed under several conditions, with the aim of comparing differences in sensitivity between strains.

In general, a dose-depended significant inhibition of swimming capacity was observed for all stressors except chloramphenicol. IBA3 showed to be more tolerant to low and high salinities, while MRS10 was more tolerant to metals. There were no differences between strains for hydrogen peroxide. For stressors such as high salinity and cadmium chloride, the swimming inhibition was mainly due to a decrease in speed. However, in organisms exposed to copper sulfate and, especially, to hydrogen peroxide, this decrease was mainly due to high attachment, which resulted in null speed values. In further studies with behavioural endpoints, it would be interesting to analyse effects not only on swimming speed, but also in swimming impairment by calculating attachment rates.

Overall, results obtained for swimming capacity did not reflect the ones observed in survival. Similar tendency between lethal and sub-lethal tests was only observed for low salinity, being IBA3 more tolerant in both situations. For both metals studied, MRS10 was more sensitive in terms of survival, but more tolerant when evaluating swimming capacity. High salinity had the same effect on strains’ survival, but lower effect in IBA3 behaviour; while MRS10 showed to be more tolerant to hydrogen peroxide at the survival level, but no differences in tolerance were observed in behaviour for this stressor. Although there are no genetic and morphological differences between strains, MRS10 strain starts reproducing at smaller sizes than IBA3 strain, and IBA3 has a longer life span (Granada et al. 2022). Foley et al. (2019) indicated several studies, including with invertebrates, where lifespan and stress resistance have shown to be positively related, and studies where extended lifespan and antioxidant capacity are strongly associated. This may explain the fact that IBA3 was, in general, more tolerant than MRS10.

Contrariwise to the other stressors, the lethal endpoint was more sensitive than the swimming behaviour after exposure to cadmium chloride (Table 2), with MRS10 showing a considerably higher value of 6 h-EC_50_ (272.14 mg L^−1^ vs 130.8 mg L^−1^), and a hormetic response (Fig. 3). This type of response was also observed for hydrogen peroxide for both strains, meaning that at low toxicity levels, rotifers were overcompensating the homeostasis disruption, presenting a response curve with a low-dose stimulation (Liang et al. 2018). This type of response occurs with a broad range of chemicals (Calabrese and Baldwin 1998), and was already observed in rotifers, more commonly when exposed to essential elements (Rebolledo et al. 2020).

Swimming behaviour showed to be especially sensitive to assess the effect of high salinity, hydrogen peroxide, and copper sulfate. As most of marine invertebrates, *B. plicatilis* has been considered an osmoconformer, meaning it keeps internal fluids isotonic to the environment (Fielder et al. 2000; Talley and Talley 2008). However, Lowe et al. (2005) observed patterns of Na^+^/K^+^ ATPase activity in *B. plicatilis* in response to salinity, suggestive of osmoregulation, being the expend of more energy on the osmotic regulation an explanation for the fact that at higher salinities (30–60 psu), in different studies, a decrease in growth rate and amictic egg production was observed (Lowe et al. 2005; Montero-Pau et al. 2011). This could explain why, at high salinities (40–80 psu), swimming capacity tests had to be performed with lower values than acute tests, as possible to observe by the difference between 24 h-LC_50_ and 6 h-EC_50_ values (Tables 1 and 2), especially for MRS10 (70.13 psu vs 49.55 psu). For hydrogen peroxide, the lowest concentration used for 24 h lethal tests was used as the highest concentration for 6 h sub-lethal exposure. And for copper sulfate, it was possible to observe sub-lethal effects at concentrations ten times lower than in the lethal test (0.0877 mg L^−1^ vs 0.81 mg L^−1^, respectively, for IBA3). Likewise, Garaventa et al. (2010) could detect alterations in swimming speed of *B. plicatilis* when exposed to concentrations that were less than 5% of LC_50_ values of three toxic substances; and the swimming behaviour of *B. koreanus* was significantly impacted by a disinfectant in concentrations lower than the lethal NOEC values (Won et al. 2022).

Chronic tests have been proposed as alternatives to lethal tests in rotifers, due to their ecological relevance (Rebolledo et al. 2020), with emphasis on behaviour endpoints (e.g. swimming behaviour), as they allow a faster assessment of effects (Dahms et al. 2011). Moreover, Won et al. (2022) showed swimming behaviour was not only more sensitive than lethal exposures, but also more sensitive than other important and more time consuming chronic endpoints, such as life table parameters. When exposing *B. koreanus* to concentrations lower than the lethal NOEC values of one toxic substance, these authors observed alterations in both swimming speed and movement patterns of rotifers, while the same conditions did not significantly changed life table parameters (Won et al. 2022). Likewise, in the present study, swimming behaviour was a good alternative to lethal endpoints to assess the effect of several stressors in rotifers. Moreover, it was possible to detect tolerance differences between strains for several types of stressors, with shorter exposures than in lethal tests and, for some of the stressors, with lower concentrations.

## Conclusion

In this study, two strains of *B. koreanus* were exposed to several abiotic stressors. The fact that one strain was generally more tolerant than the other, although they belong to the same species and are maintained under the same conditions for several years, evidenced the importance of knowing the characteristics of the water body of origin and to perform multiclonal experiments. The results also highlighted the potential of swimming capacity inhibition as a toxicity endpoint to assess differences in response between rotifer strains, being more sensitive and of shorter duration than lethal assays.

## Data Availability

The data that support the findings of this study are available from the corresponding author, upon reasonable request.
